# The Role of Protein and Fat Intake on Insulin Therapy in Glycaemic Control of Paediatric Type 1 Diabetes: A Systematic Review and Research Gaps

**DOI:** 10.3390/nu13103558

**Published:** 2021-10-11

**Authors:** Dieter Furthner, Andreas Lukas, Anna Maria Schneider, Katharina Mörwald, Katharina Maruszczak, Petra Gombos, Julian Gomahr, Claudia Steigleder-Schweiger, Daniel Weghuber, Thomas Pixner

**Affiliations:** 1 Department of Paediatric and Adolescent Medicine, Salzkammergutklinikum Voecklabruck, 4840 Voecklabruck, Austria; dieter.furthner@ooeg.at (D.F.); andreas.lukas@ooeg.at (A.L.); thomas.pixner@ooeg.at (T.P.); 2 Obesity Research Unit, Paracelsus Medical University, 5020 Salzburg, Austria; mar.schneider@salk.at (A.M.S.); k.moerwald@salk.at (K.M.); katharina.maruszczak@aon.at (K.M.); j.gomahr@salk.at (J.G.); 3 Department of Paediatrics, Paracelsus Medical University, 5020 Salzburg, Austria; c.steigleder-schweiger@salk.at; 4 Department of Paediatric and Adolescent Surgery, Paracelsus Medical University, 5020 Salzburg, Austria; p.gombos@salk.at

**Keywords:** diabetes, type 1 diabetes mellitus, children, fat, protein, nutrition, high fat, high protein, mixed meal, paediatric, insulin

## Abstract

Carbohydrate counting (CHC) is the established form of calculating bolus insulin for meals in children with type 1 diabetes (T1DM). With the widespread use of continuous glucose monitoring (CGM) observation time has become gapless. Recently, the impact of fat, protein and not only carbohydrates on prolonged postprandial hyperglycaemia have become more evident to patients and health-care professionals alike. However, there is no unified recommendation on how to calculate and best administer additional bolus insulin for these two macronutrients. The aim of this review is to investigate: the scientific evidence of how dietary fat and protein influence postprandial glucose levels; current recommendations on the adjustment of bolus insulin; and algorithms for insulin application in children with T1DM. A PubMed search for all articles addressing the role of fat and protein in paediatric (sub-)populations (<18 years old) and a mixed age population (paediatric and adult) with T1DM published in the last 10 years was performed. Conclusion: Only a small number of studies with a very low number of participants and high degree of heterogeneity was identified. While all studies concluded that additional bolus insulin for (high) fat and (high) protein is necessary, no consensus on when dietary fat and/or protein should be taken into calculation and no unified algorithm for insulin therapy in this context exists. A prolonged postprandial observation time is necessary to improve individual metabolic control. Further studies focusing on a stratified paediatric population to create a safe and effective algorithm, taking fat and protein into account, are necessary.

## 1. Introduction

Worldwide the prevalence of children with Type 1 Diabetes mellitus (T1DM) is increasing [1]. The current cornerstones of T1DM therapy include lifetime management of exogenous insulin delivery, dietary and exercise management [2]. Insulin pump therapy and continuous glucose monitoring (CGM) represent the current technical state of the art of insulin management and have been shown to be associated with a reduction of cardiovascular comorbidities (CVCM) [3]. Given the availability of 24/7 glucose monitoring, health care professionals have become more aware of the effects of all macronutrients on prolonged postprandial glucose levels. In fact, optimal postprandial glucose levels depend on matching insulin to the macronutrient meal composition. However, although there is evidence that fat and protein influence insulin requirement of children with T1DM, current recommendations are still solely based on meal-based carbohydrate content [4]. The aim of this review is to summarize the current evidence of the effects of dietary fat and protein in children with T1DM on prandial insulin requirements.

## 2. Background

### 2.1. Epidemiology

T1DM is the main type of diabetes in children and adolescents with a rise in prevalence and incidence [4,5]. It is currently estimated that each year 96,000 children under the age of 15 develop T1DM worldwide [6]. T1DM incidences show a great variability between different countries as well as between ethnic populations [7]. In addition, the prevalence rates for overweight/obesity in children with T1DM at least parallels the worldwide increase in the general paediatric population [8,9], which highlights the particular importance of healthy nutrition including the role of macronutrients in this subgroup.

### 2.2. Treatment of T1DM

The current therapy of T1DM in children is based on three cornerstones: insulin replacement, physical activity, and nutrition.

#### 2.2.1. Insulin and Monitoring

Insulin replacement should mimic physiological patterns including baseline as well as bolus insulin for meals and in hyperglycaemia. It can be conducted either by multiple daily injections (MDI) or as a continuous subcutaneous insulin infusion (CSII). Mealtime bolus insulin is commonly calculated by counting carbohydrates (CHC). CHC is defined as how much insulin is given per 10 g of carbohydrates [4]. The International Society for Paediatric and Adolescent Diabetes (ISPAD) guidelines state that fat and protein should be considered for determining the insulin bolus dose and delivery (e.g., dual bolus), yet do not provide any detailed recommendations [4].

Methods that not solely take carbohydrates into consideration for calculating bolus insulin are the Food Insulin Index (FII) [10] and the Pankowska Index/equation (additional 1 U of insulin for every 100 kcal of fat or protein) [11]. While the FII is based on the total calorific sum of a meal given by a reference list, the Pankowska equation converts the kcal of fat and protein into carbohydrate units [11]. However, these are not routinely used in everyday life.

Home self-monitoring of glucose levels is possible by finger stick or tissue glucose monitoring, which is also known as continuous glucose monitoring (CGM). CGM is nowadays commonly paired with CSII. The invention and establishment of CGM has resulted in improved levels of HbA1c [12,13]. The usage of CGM allows for continuous measurements instead of single point measurements. The definition of area under the curve (AUC) for glucose levels allows calculation of euglycemic time in target range (also called time in range (TIR)) [14]. This AUC/TIR via CGM is already complementing and will probably supersede HbA1c in the future in patients with CGM, as HbA1c only reflects glycemia over the previous 4 to 12 weeks and is unable to provide information on daily and postprandial glucose levels [15]. Since its introduction in everyday use, CGM has put a focus on the composition of meals and how macronutrients (protein, fat and carbohydrates) influence and shape glycaemic curves [16].

#### 2.2.2. Nutrition

A healthy and balanced diet is recommended for the entire paediatric T1DM population. Nutritional education should consider the patient’s cognitive and psychosocial behaviour and should be appropriate for culture, ethnic background, and family tradition. Daily calorific intake and the distribution of macronutrients should focus on maintaining ideal body weight, optimize growth and development. According to ISPAD guidelines, carbohydrates should cover 45–55%, fat 30–35% and protein 15–20% of the daily energy intake [4]. A high total intake of fat is linked to an increased risk of being overweight and obesity [17]. A protein intake of 15% to 20% of total daily energy dose is equal to 2 g/kg/d in early infancy, 1 g/kg/d for a 10-year-old and 0.8–0.9 g/kg/d in later adolescence [18]. However, these goals are irregularly met, especially with a surplus of consumed fat [19,20]. In line with the focus of this review on fat and protein, these two macronutrients and their respective roles will be addressed in detail.

### 2.3. Physiology of Dietary Fat and Protein on Blood Glucose Levels

Dietary fat and protein have been recognized to significantly elevate postprandial blood glucose levels [21]. The mechanisms by which dietary fat influences blood glucose levels include direct effects (free fatty acids (FFAs) stimulate pancreatic beta cells and insulin secretion), effects on other hormones (release of glucagon, glucagon-like-protein 1 (GLP-1), gastric inhibitor polypeptide (GIP) and ghrelin), gastric emptying (additional fat as part of a meal delays gastric emptying) and gluconeogenesis [22,23]. Dietary protein elevates blood glucose levels by alteration of the hormones affecting glucose homeostasis (high protein meals induce elevated plasma glucagon levels, cortisol, growth hormone, insulin-like-growth-factor 1 (IGF-1) and ghrelin) and gluconeogenesis [22]. Dietary fat, added to carbohydrates results in an initially reduced glycaemic postprandial response (first 1–3 h (h)), due to delayed gastric emptying. This extends and increases the glycaemic response over multiple hours [22].

In non-diabetic individuals, dietary protein does not elevate postprandial blood glucose levels [24]. In contrast, the addition of protein to carbohydrates in diabetic patients leads to elevated blood glucose levels and insulin requirements. Several studies investigated the influence of protein on postprandial glycaemic response. Paterson et al. showed that adding ≥28 g protein to a mixed meal or consuming ≥75 g of protein by itself leads to significant and prolonged postprandial hyperglycaemia in children and young adults aged 7 to 40 years [25]. Hyperglycaemia was demonstrated to start 2–3 h postprandially and last at least 5 h [25,26]. The effect of dietary fat and protein influences blood glucose levels individually but is also accumulative. This reflects the composition of our diet that usually combines all three macronutrients [21,22].

## 3. Methods

We performed a literature search using the electronic database Pubmed in accordance with the Preferred Reporting Items for Systematic Reviews and Meta-Analyses (PRISMA) methodology.

**Figure 1:**
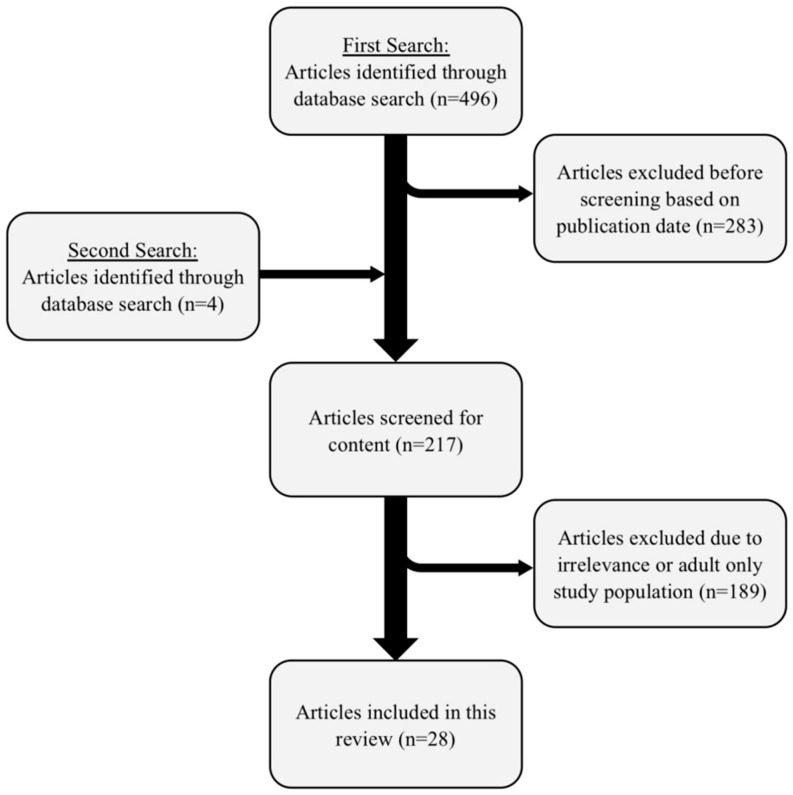
Graphic display of literature search using the electronic database Pubmed in accordance with the Preferred Reporting Items for Systematic Reviews and Meta-Analyses (PRISMA) methodology.

The search parameters included original articles or reviews about patients (any sex, age, race, or comorbidity were included). The search was limited to articles published between 1 January 2011 and 1 May 2021. The first search without filters was performed relating to ‘‘type 1 diabetes” and ‘‘protein” and ‘‘fat” and ‘‘insulin”. This resulted in a total of 496 articles. Non-English publications were excluded. For the purpose of this review, the paediatric population was defined as being under the age of 18 years old. This search resulted in a total of 213 articles (496 − 283 = 213). In an additional search the parameters “ISPAD” and “guideline” and “nutrition” were searched for, resulting in four additional original articles, reviews, and guidelines (see Figure 1). This left 217 articles.

Two of the authors (TP, DF) reviewed the titles and abstracts for articles with a paediatric population with type 1 diabetes that focused on high-fat and/or high-protein meals, postprandial glucose levels and insulin and excluded articles deemed irrelevant or with a study population exclusively 18 years and older.

After exclusion of entirely adult study populations and non-relevant publications (n = 189), a total of 27 available articles and the ISPAD guidelines [4] remained for analysis (see Table 1 and Table 2).

**Table 1:** Characteristics of reviewed original articles between 1 January 2011 and 1 May 2021 with effect of dietary fat and protein for prandial insulin requirements in children with Type 1 Diabetes.

Authors and Year	Sample Size	Average Age ± SD in Years (Range of Age)	BMI z-Score	HbA1c (% or mmol/mol)	Meal Type (HF/HP/Mixed Meal)	Composition of the Test Meals (Carbohydrates/Fat/Protein in g)	Measurement Methods/Duration of Observation	Insulin Regimen (Absolute/%)	Algorithm for Calculating Bolus Insulin
**Paediatric Study Population Only**									
Seckold R et al., 2019 [19]	22	4.9 ± 1.3(2.5 to 6.6)	z-Score 0.8 ± 0.9	6.4% ± 0.9% 47 ± 10 mmol/mol	retrospective 3-day meal observation via questionnaire			CSII 41%, MDI 59%	CHC
Katz ML et al., 2014 [20]	252	13.2 ± 2.8 (8 to18)	z-Score 0.7 ± 0.8	8.51% ± 1.3%	retrospective 3-day meal observation via questionnaire			CSII 69%, MDI 31%	no data on specific algorithms
Smart CE et al., 2013 [21]	33	12.2 ± 2.5 (8 to 17)	z-Score 0.6 ± 0.8	7.2% ± 0.8%	LFLP//LFHP//HFLP//HFHP	LFLP 30.3 g/4 g/5.3 g//LFHP 30 g/3.9/40 g//HFLP 30.3 g/35 g/5.3 g//HFHP 29.8 g/35.2 g/40 g	CGM/5 h	CSII n = 27, MDI n = 6	CHC
van der Hoogt M et al., 2017 [27]	22	10.4 ± 4 (4 to 17)	z-Score −1–+3	8.23% ± 0.82%	LFLP//HFHP	individually calculated total daily energy requirement using age/weight/gender: LFLP 60%/25%/15%//HFHP 40%/35%25% LFLP 40.2 g fat (±9.08)/7.72 (±2.25)/10.6 (±3.37) 10.6//HFHP 40.2 (±9.08)/15.3 (±4.03)/26.6 (±6.72)	CGM + cap/10 h	CSII	CHC
Abdou M et al., 2021 [28]	51	11.24 ± 2.41 (6 to 18)	no data	8.35% ± 0.99%	mixed meal//HP//HF	25% daily caloric intake//HP (+125 kcal Protein)//HF (+125 kcal Protein)	cap/5 h	MDI	CHC
Kaya N et al., 2020 [29]	30	16 (16 to 18)	z-Score −0.2	7.6% (6–11.2%)	mixed meal//HP//HFHPa//HFHPb(mit Pankowska)	meals were age adjusted: 25% of the total daily energy requirement—mixed meal 70 g/17 g/26 g//HP 70 g/26 g/36 g//HFHPa 70 g/30 g/36 g//HFHPb 70 g/30 g/36 g	cap/4 h	MDI	CHC and Pankowska Equation
Piechowiak K et al., 2017 [30]	58	14.7 ± 2.2 (10.5 to 18.0)	z-Score 0.3 ± 1.1 (BMI 21.5 ± 3.6)	8.3% ± 11% 67.2 ± 12 mmol/mol	LFHP	30 g/5 g/36 g	CGM + cap/3 h	CSII	CHC and Pankowska Equation
Lopez PE et al., 2018 [10]	33	12.3 ± 3.6 (7 to 17)	z-Score 0.2 ± 1.0	7.3% ± 0.7%	HF//HP	47 g/27 g/16 g//48 g/13 g/34 g	CGM/5 h	CSII	CHC and Pankowska Equation and Food Index
Pankowska E et al., 2012 [31]	24	12.7 to 17.9	z-Score 0.7 (−1.1–0.98)	7.5% ± 1.3% (5.1–9.9%)	mixed meal (Pizza)	46.8 g/33.1 g/25.4 g	cap/6 h	CSII	CHC and Pankowska Equation

BMI: Body Mass Index (in kg/m^2^), SD: standard deviation, HbA1c: glycosylated haemoglobin concentration (in % or mmol/mol), HF: high fat, HP: high protein, LF: low fat, LP: low protein, LFLP: low fat low protein, HFLP: high fat low protein, HFHP: high fat high protein, LFHP: low fat high protein, Measurement methods: cap: capillary blood sample, CGM: continuous glucose monitoring, CSII: continuous subcutaneous insulin infusion, MDI: multiple daily injection, CHC: carbohydrate counting.

**Table 2:** Reviews for mixed study population between 1 January 2011 and 1 May 2021.

Authors and Year	Sample Size	Average Age ± SD in Years (Range of Age)	BMI z-Score	HbA1c (% or mmol/mol)	Meal Type (HF/HP/Mixed Meal)	Composition of the Test Meals (Carbohydrates/Fat/Protein in g)	Measurement Methods/Duration of Observation	Insulin Regimen (Absolute/%)	Algorithm for Calculating Bolus Insulin
**Mixed Study Population (Children And Adults)**									
Neu A et al., 2015 [32]	15	16.8 ± 2.9	BMI 21.1 ± 2.19	6.9% ± 0.8	mixed meal//HFHP	70 g/19 g/28 g//70 g/52 g/110 g	CGM/12 h	CSII 6, MDI 9	CHC
Evans M et al., 2019 [33]	11	16.5 ± 2.7 (12 to 21)	z-Score 0.4 ± 0.6	6.9% ± 0.8 52 ± 8.7 mmol/mol	HP//LP	31 g/8 g/60 g//31 g/8 g/5 g	Insulin clamp variation, cap/5 h	Intravenous Insulin infusion to maintain euglycaemia	Pankowska Equation
Lopez PE et al., 2017 [34]	19	12.9 ± 6.7 (6.2 to 19.6)	z- Score 0.4 ± 0.7	6.9% ± 0.6	HFHP	30 g/35 g/40 g	CGM/5 h	CSII	CHC
Kordonouri O et al., 2012 [35]	42	12.3 ± 3.6 (6 to 21)	no data	no data	mixed meal (Pizza)	Pizza—50% carbohydrate, 34% fat, 16% protein—corresponding to 33%of age-adjusted daily energy requirement	cap/6 h	CSII, normal and dual-wave bolus	CHC and Pankowska Equation
Paterson MA et al., 2016 [25]	27	21.7 ± 11.7 (7 to 40)	BMI 21 ± 3.1	6.9% ± 0.8 52 ± 9.1 mmol/mol	2× carbohydrates only//LP to HP	10 g/0 g/0 g//20 g/0 g/0 g//0 g/0 g/0 g//0 g/0 g/12.5 g//0 g/0 g/25 g //0 g/0 g/50 g//0 g/0 g/75 g//0 g/0 g/100 g	CGM/5 h	CSII 14, MDI 12	CHC
Schweizer R et al., 2020 [36]	16	18.2 ± 2.8 (15.2 to 24)	no data	7.15% (6.2–8.3%)	mixed meal//HFHP	70 g/19 g/28 g//57 g/39 g/92 g	CGM/12 h	CSII 10, MDI 6	CHC +20% and +40% extra insulin for fat and protein
Smith TA et al., 2021 [37]	24	19 ± 9 (9 to 35)	BMI 20.9 (children) BMI 24.6 (adults)	6.7% ± 0.7 49 ± 8 mmol/mol	HFHP	30 g/40 g/50 g	CGM/5 h	MDI	CHC
Paterson M et al., 2020 [38]	26	21.7 ± 8.14 (8 to 40)	BMI 22 ± 3.6	6.9% ± 0.6 52 ± 9.1 mmol/mol	LFHP	30 g/<1 g/50 g	CGM/4 h	CSII	CHCand Pankowska Equation
Smith TA et al., 2021 [39]	27	15 ± 4 (10 to 23)	BMI 21.3 (children) BMI 24.6 (adults)	7.0% ± 0.7 53 ± 7 mmol/mol	HFHP	30 g/40 g/50 g	CGM/5 h	CSII	CHC
Paterson MA et al., 2017 [40]	27	20.7+/−10.3 (10 to 40)	BMI 22 ± 3.6	7.1% ± 0.95 54 ± 3.1 mmol/mol	LFLP//LFHP	30 g/0.4 g/0–12.5–25–50–75 g	CGM/4 h	CSII 16, MDI 11	CHC
De Palma A. et al., 2011 [41]	38	6 to 19	BMI 21.9 ± 4.3	7.66% ± 0.81	mixed meal (Pizza)	carbohydrate 60%, fat 23%, protein 16%; 35% of total daily caloric intake	cap/6 h	CSII	CHC

BMI: Body Mass Index (in kg/m^2^), SD: standard deviation, HbA1c: glycosylated haemoglobin concentration (in % or mmol/mol), HF: high fat, HP: high protein, LF: low fat, LP: low protein, LFLP: low fat low protein, HFLP: high fat low protein, HFHP: high fat high protein, LFHP: low fat high protein, Measurement methods: cap: capillary blood sample, CGM: continuous glucose monitoring, CSII: continuous subcutaneous insulin infusion, MDI: multiple daily injection, CHC: carbohydrate counting.

Out of the 20 original articles, nine publications [10,19,20,21,27,28,29,30,31] investigated a paediatric population (<18 years old) only with a total number of 525 participants. Two of these articles (total number of 274 children) feature a questionnaire about dietary habits of diabetic children only and had no focus on blood sugar or bolus insulin following high fat and/or high protein meals [19,20]. This leaves 251 children in paediatric population only in therapeutic studies. Study group characteristics are given in Table 1.

Two studies that were defined as paediatric population by the original authors included patients up to the age of 19; we included these in the mixed population study group [34,41]. The youngest participant in an original therapeutic (meal based) study was 4 years old [27], while the youngest participant in a questionnaire-based study was 2.5 years old [19].

We found 11 original articles with a mixed (paediatric and adult) study population [25,32,33,34,35,36,37,38,39,40,41]. The youngest participant was 6 years old [41], the oldest was 40 years old [38]. The mixed study population included a total of 272 persons.

Table 1 and Table 2 give the age, duration of illness, BMI, HbA1c, method and duration of glucose monitoring, insulin regimes (MDI vs. CSII) and the number of participants as listed. All original articles included male and female participants with a primarily balanced gender distribution, except for Neu et al. [32] which included 13 males and two females. The results between both genders did not differ significantly. Duration of illness was reported to be at least one year in all original articles. In the paediatric population the maximum duration was 16 years [29], in the mixed population 23 years [39]. HbA1c in the paediatric population only was between 5.0% (6.4 ± 0.9%, mean ± SD) [19] and 11.2% (mean 7.6%) [29]. In the mixed population HbA1c was between 6.0% (mean ± SD 6.7 ± 0.7%) [37] and 7.66 ± 0.81% (mean ± SD) [41]. No original article reported a mean HbA1c of ≥8.5%.

Body composition as represented through body-mass-index (BMI) was not comparable as it was listed as a mixture of total values [25,32,38,39,40,41], depending on z-score [10,19,20,21,27,29,30,33] or according to percentile [31,34]. The widest range for the z-score in the paediatric population only was between −1 and +3 [27]. This study included 16 participants with normal weight, five children with a risk of becoming overweight and one overweight [27]. All Studies included children with obesity, but the mean BMI according to z-score was always around 1.0 (normal weight). In the mixed population, reporting of BMI was according to percentile [34], z-score [33] or a total number [25,32,37,38,39,40,41] or no data [35,36] was given.

## 4. Results

### 4.1. Effect of Macronutrients Fat and Protein on Glycaemic Control and Therapy

#### 4.1.1. Protein as Dominant Macronutrient

##### Glycaemic Response

Neu et al. showed peak blood glucose level after 2–3 h postprandially to their protein based standard meal (see Table 2) and a significantly increased AUC after 12 h [32]. Bell et al. reported that postprandial blood glucose is not just affected by the amount of protein but the composition of the meal’s other macronutrients as well [42]. The effect of protein varies on its combination with carbohydrates. Paterson et al. concluded that ≥75 g of protein alone significantly increases the blood glucose level between minutes 150 to 300 postprandially, while Smart et al. wrote that 30 g of protein in combination also leads to elevated glucose levels [21,40].

##### Amount of Protein Studied

ISPAD reports protein requirements of 2 g/kg/d in infancy, decreasing to 0.9 g/kg/d in later adolescence but does not give absolute numbers per meal [4]. Protein content in test-meals ranged from 5 g [21] to 100 g. In the article with 100 g of protein, a test-drink was given without age-adjustment to a population between seven and 40 years old [25]. No meal was without protein and while no definition of low protein was given, the authors’ definition of high protein was non comparable. Evans et al. defined high protein as 60 g total, while Paterson et al. defined high protein as 50 g and Smart et al. defined 40 g of protein as high [21,33,38]. Abdou et al. used a stratification according to age that defined high protein as 53 g (age 6–10 years), 61.9 g (10–14 years), 71.5 g (14–19 years) [28].

##### Insulin Therapy

Evans et al. (60 g protein) report that about 50% more insulin is necessary to maintain euglycemia after a high protein meal, with a high interindividual variability (90% to 600% total bolus insulin) [33]. Piechowiak et al. (36 g protein) used a dual wave bolus with additional insulin to improve postprandial glucose levels after a high protein meal, using CFP-based bolus insulin calculation [30].

#### 4.1.2. Fat as Predominant Macronutrient

##### Glycaemic Response

Glycaemic responses to high fat meals (and low protein) have been described as initially reducing glucose levels within the first 1–2 h, followed by elevated levels of up to 5 h [21,25]. This is explained by delayed gastric emptying, inducing gluconeogenesis, direct effect of free fatty acids and the influence of fat on hormones [28]. The two high fat (and low protein) articles contradict these findings. Abdou et al. reported glucose peak levels at 2 h postprandially with a normalization towards 5 h (insulin was based on CHC) [28]. Lopez et al. documented the peak glucose excursion between 120 and 180 min after a high fat meal depending on the bolus insulin calculation algorithm (CHC, FII, Pankowska) but without significant difference between the blood glucose levels [10].

##### Amount of Fat Studied

ISPAD recommends a fat intake of 30–35% of the total daily energy intake [4]. While this represents the daily distribution, it does not reflect the individual meal level. Fat content in test meals ranged from 0 g [25] to 52 g [32]. All authors focusing on fat as the predominant macronutrient except Van der Hoogt [27] defined their non age-adjusted meal as high fat with a fat content of 30 g or higher, but not exceeding 52 g [21,27,28,29,32,34,36,37,39]. Van der Hoogt et al. used a HFHP meal in a paediatric population (4–17 years old) and an age-adjusted meal with a meal fat content of 15.3 g ± 4.03 g (mean ± SD) [27]. There were two studies that included a high fat meal only as part of the dietary regimen [10,28]. In the other articles fat was administered in a combination meal with varying carbohydrate and protein content. No study with low fat or high fat only (i.e., without any protein) was found (see Table 1 and Table 2).

##### Insulin Therapy

Lopez et al. reported 17% additional bolus insulin for a high fat meal, based on Pankowska equation, than based on CHC, resulting in a better glycaemic profile but with a higher rate of hypoglycaemia [10]. Wolpert et al. noted that fifty grams of fat can double the insulin requirements but interindividual differences in the glycaemic response were noted [43].

#### 4.1.3. Combination of High Fat and High Protein in Meals

##### Glycaemic Response

High fat and high protein meals resulted in an additive effect with a delayed postprandial glycaemic elevation. Smart et al. found hyperglycaemia from 3 to 5 h after the meal [21]. Van der Hoogt et al. observed hyperglycaemia up to 8.5 h (total observation time was 10 h) [27]. Neu et al. even observed 12 h of hyperglycaemia [32].

##### Amount of High Fat and High Protein Studied

Please see Table 1 and Table 2.

##### Insulin Therapy

Additional insulin for a HFHP combination was necessary and dosage increased with the duration of illness [27]. Lopez concluded that combination bolus 70%/30% compared to a standard bolus resulted in significantly lowered AUC [34]. Smith et al. recommended 40% additional bolus insulin in CSII. In a different study Smith et al. recommended 125% of CHC-calculated bolus insulin in MDI [37,39]. Neu et al. point out that a diurnal variation in insulin sensitivity may influence bolus insulin requirements [32]. Authors concluded that calculation based solely on CHC was insufficient for maintaining postprandial euglycaemia. Taking high fat and high protein into account for calculating bolus insulin was a common recommendation [21,27,29,32,34,37,39] echoing the ISPAD clinical guidelines [4]. Schweitzer et al. suggested that the introduction of a protein unit (50 g protein) equalling 1 carbohydrate unit (10 g carbohydrates) was necessary. They do not recommend taking high fat into account, quoting articles by Peters and Nordt [36,44,45]. Van der Hoogt found that high fat (15 g in a test meal), high protein content (26 g in a test meal) required an average of eight times more postprandial correction insulin than in low fat (7 g in a test meal), low protein meals (10.6 g in a test meal) [27].

#### 4.1.4. Meal Adjustment According to Age and/or Weight

Meal composition between studies was non comparable. In the articles with a paediatric population four publications adjusted the test meals according to age and/or weight [21,27,28,29]. Three publications were not including test meals [4,19,46] and 6 articles [10,20,30,32,33,34] did not adjust the test meals according the age and/or weight.

In the 11 articles with a mixed (paediatric and adult) study population (see Table 2) there were two publications that adjusted the test meals according to age and/or weight. These articles featured a study population of up to 19 [41] and 21 years [35]. Six articles of the mixed population did not specify a test meal [22,42,47,48,49,50] and 6 did not adjust the test meals [25,36,37,38,39,40]. Table 1 and Table 2 list the meal compositions used in the articles mentioned.

### 4.2. Insulin Therapy

#### 4.2.1. Counting of Macronutrients: CHC vs. CFP (Carbohydrate-Fat-Protein)

Eleven papers based the calculation of bolus insulin for meals on the individualized, standardized CHC, regardless of the meal composition (four paediatric only [19,21,27,28], seven mixed population [25,32,34,37,39,40,41]). Eight articles focused on calculating bolus insulin dose depending on fat and/or protein in addition to CHC (four paediatric only [10,29,30,31], four mixed population [33,35,36,38]).

The methods used for calculating additional protein and fat were the Pankowska Index/equation [10,29,30,31,33,35,38] as well as the Food Insulin Index (FII) [10]. Lopez et al. found that FII was not better than CHC to manage postprandial glycaemic excursions [10]. Piechowiak et al. used the term fat-protein exchange (1 fat-protein exchange 100 kcal for protein and fat equaling 40 kcal of carbohydrates) mimicking the Pankowska equation [30]. In the original articles by Pankowska, no hypoglycaemia was mentioned [11,51]. In contrast, in four other articles, use of the Pankowska equation resulted in an improved postprandial glycaemic profile, but resulted in significantly more hypoglycaemic events [10,30,35,38]. Kordonouri et al. found significantly more hypoglycaemic events (35.7% vs. 9.5%) when using the Pankowska equation compared to CHC in a 6 h observation period [35]. Lopez et al. argue that a longer observation time (6 h) compared to Pankowska’s (2 h) [51] led to the discovery of more hypoglycaemic events [10]. Further, Schweitzer et al. used an individual calculational approach, suggesting that beside carbohydrates only protein and not fat should be taken into calculation (“protein unit”) with 50 g of protein equalling 10 g of carbohydrates for extra insulin [36].

#### 4.2.2. Amount of Bolus Insulin for Covering Fat and/or Protein

Only one study was performed using a modified intravenous insulin clamp technique in 11 patients, aged 12 to 21 years (16.5 ± 2.7, mean ± SD) and focused on dietary protein only. The investigators found that high protein meals require about 50% more insulin to maintain euglycemia than a low protein meal that contains the same amount of carbohydrates. The majority (60%) of bolus insulin was required within the first two hours. Large interindividual differences (−1.3 to 9.4 units) of bolus insulin were described [33]. There were no clamp studies focusing on high fat or the combination of high fat and high protein.

Three articles in a paediatric-only population administered insulin based on CHC only with meals that included a varying amount of fat and protein. No additional insulin was added based on calculation (e.g., +20% of additional insulin) or fat and protein content. Elevated and prolonged blood glucose levels were observed, and it was concluded that additional insulin for fat and protein is necessary as well as longer postprandial observation time [21,27,28]. Similar studies exist for a mixed age population with equal results [32,40,41].

Four articles investigated increased insulin doses. Based on CHC, participants’ individual insulin: carbohydrate ratios were used to calculate insulin (100%) and then adding insulin (e.g., +20%, +40%). No study administered more than 160% based on CHC [36,37,38,39].

Paterson et al. argued that 60% additional insulin was the upper limit, as the Pankowska equation would lead to comparable additional insulin. The authors concluded that 30% additional insulin, delivered via combination bolus, results in improved postprandial blood glucose without an increased risk of hypoglycaemia [38].

Schweitzer et al. used CHC for various meals (carbohydrate only, high-fat-high-protein (HFHP), standard meal) and added different amounts of additional insulin (+20%, +40%) to cover fat and protein. This article concludes that the AUC for glucose in the observed time for fat- and protein-rich meals without additional insulin was significantly higher (1968 ± 581 mg/dL/12 h) than with additional insulin (+20% 1603 ± 561 mg/dL/12 h; +40% 1527 ± 461 mg/dL/12 h). The authors suggest that only protein and not fat should be taken into calculation with 50 g of protein equalling 10 g of carbohydrates [36]. Smith et al. compared single- vs. split-bolus, insulins (aspart vs. regular) and insulin dose (100% vs. 125%) in an MDI regimen. They concluded that 25% additional aspart-insulin for a HFHP breakfast significantly improved postprandial glycemia without hypoglycaemia [37].

In a different study Smith et al. increased the insulin dose from 100% to 140% and 160% for a HFHP breakfast and found that 140% of calculated insulin based on CHC, administered as a combination bolus via CSII, improved the postprandial AUC without increasing hypoglycaemia, which was at higher risk at 160% [39]. These results are in accordance with previous results that state, that using the Pankowska equation (equalling around 160% insulin compared to CHC) leads an increased risk of hypoglycaemia [10,30,35,38].

#### 4.2.3. Ways of Administering Insulin (MDI vs. CSII)

Table 1 and Table 2 display the ways in which insulin was administered (MDI vs. CSII).

#### 4.2.4. Choice of Bolus Type in CSII and MDI

Piechowiak et al. compared different bolus algorithms for CSII (normal-dual vs. dual-normal bolus). Bolus insulin was calculated by CHC and adding additional insulin for protein (one fat-protein exchange 100 kcal for protein and fat equaling 40 kcal of carbohydrates) in 58 children aged 10.5–18 years (14.7 ± 2.2 years). This study contained high protein, low fat meals only. A dual wave bolus for high protein and additional insulin for protein gave the best results (finger prick blood glucose) in the 3 h observation time. Mean blood glucose level after 180 min postprandially was 123 ± 43.18 mg/dL with a standard bolus and no additional insulin as compared to a dual-wave bolus 87.15 ± 38.74 mg/dL [30].

Paterson et al. investigated various amounts of insulin, using a combination bolus (65% of the standard dose given up front) for a study comparing the blood glucose elevation after a breakfast drink (50 g protein, 30 g carbohydrate, 0.3 g fat). The study included 26 patients, age 8–40 years (21.7 ± 8.14, mean ± SD). Observation time was 4 h, using CGM and CHC. The authors concluded that an additional 30% of insulin resulted in the best result and almost return to baseline after 4 h without increased risk of hypoglycaemia (higher risk at 145% and 160% bolus insulin), based on CHC calculation. Authors recommend adding 30% of insulin for a high protein (≥50 g) meal [38].

Lopez et al. investigated five different combination boli in patients with CSII in comparison to their individual standard bolus. They concluded that for a high fat and high protein meal additional insulin of up to 70% of the insulin:carbohydrate ratio in the extended bolus is needed to maintain euglycemia. A standard bolus based on CHC was only able to provide blood sugar control within the first 120 min, resulting in progressive elevation of blood glucose levels afterwards until the end of observation at 300 min. A combination bolus of ≥60% of the insulin:carbohydrate ratio was required in order to control postprandial blood glucose elevation [34].

Kordonouri et al. performed a study on 42 patients aged 6–21 years (12.3 ± 3.6, mean ± SD), using sensor-augmented-pumps. Glucose profiles over a period of 6 h postprandially to a standardized pizza meal were obtained. CHC only resulted in a significantly higher AUC (926 ± 285 mg/dL × 6 h) and average glucose level (160.5 ± 51.9 mg/dL) as compared to taking supplementary fat and protein into account (AUC: 805 ± 261 mg/dL × 6 h, average glucose 137.8 ± 46.2 nmg/dL). The type of bolus setting (normal vs. dual-wave bolus) made no difference. At the end of the observation period, pre-prandial glucose levels were not reached, with the standard bolus and CHC resulting in the longest time of hyperglycaemia [35].

De Palma et al. investigated a simple bolus versus a double wave bolus (30/70) extended over a 6 h period administered given either immediately or 15 min before a pizza meal. The study population included 38 patients, aged 6–19. Observation period was 6 h via finger prick glucose measurements. The study was based on CHC and found that a simple bolus given 15 min before the meal led to best results (simple bolus 15 min before meal: AUC 6.9 ± 14.9 mg/dL/min × 10^3^; simple bolus immediately before meal: AUC 4.2 ± 25.9 mg/dL/min × 10^3^; double-wave bolus given 15 min before the meal AUC 1.9 ± 21.3 mg/dL/min × 10^3^; double-wave bolus given immediately before the meal AUC 13.3 ± 15.6 mg/dL/min × 10^3^) [41].

Smith et al. investigated standard vs. split bolus based on CHC and MDI in a population of 24 patients, aged 9–35 years (19 ± 9, mean ± SD) after a high fat, high protein meal (40 g fat, 50 g protein, 30 g carbohydrates). Observation time was 5 h and glucose levels were monitored using CGM. Baseline glucose levels were achieved with 125% of a standard bolus, resulting in a significantly better AUC (341 ± 169.512 mmol/L × min) compared to 100% insulin (AUC 620 ± 451.788 mmol/L × min; *p*-value 0.016). A split bolus resulted in no glycaemic benefit [37].

#### 4.2.5. Interindividual Variation of Insulin Therapy

Four papers highlighted interindividual variations of results and advocated the need for an individualized insulin therapy. Authors stated that a unified recommendation was difficult because of individual glycaemic response to protein intake, individual insulin resistance and duration of illness [10,28,30,33].

## 5. Discussion

Type 1 Diabetes mellitus is a disease that affects around 1.1 million children and adolescents <20 years worldwide. These numbers are on the increase [6]. The number of studies in the last 10 years (n = 28) is limited as is the maximum size of the study populations (n = 58 for paediatric only [30] vs. n = 42 in a mixed population [35]) after excluding retrospective questionnaire-based articles. Study populations were heterogenous concerning age, treatment, and study parameters.

### 5.1. Effect of Fat and Protein on Glucose Response

ISPAD recommends taking fat and protein into account when calculating bolus insulin but gives neither a threshold for these macronutrients nor a specific insulin dosage algorithm but refers to a number of reviewed articles [4,21,31,43,52,53].

#### 5.1.1. Fat

In the course of research, the definition of high fat was a dietary recommendation concerning daily intake at 30–35% but no definition per single meal [4]. All author focused on fat as a predominant nutrient, but Van der Hoogt et al. [27] used 30 g and more as a definition of high fat, with a maximum of 52 g [21,27,28,29,32,34,36,37,39]. Van der Hoogt et al. defined 15.3 g ± 4.03 g (mean ± SD) in their age adjusted meal as high fat [27]. Except for two high fat only studies, all fat was part of a mixed meal [10,28]. Authors do not declare the type of fat (e.g., triacylglycerols).

Glycaemic responses to nutritional fat were reported in the results section with partly contradictive results (see above). What remains is a prolonged hyperglycaemia after ingestion of nutritional fat that requires a prolonged monitoring (e.g., CGM) and prolonged dispense of insulin. As noted in the results section fifty grams of fat can double the insulin requirements but interindividual differences in the glycaemic response were noted [43]. The role of fat as a single macronutrient remains controversial as Peters and Davidson stated that fat does not increase the postprandial glucose response. They argue that in non-study settings high fat meals are often conjugated with high carbohydrate contents hence resulting in postprandial hyperglycaemia [44]. In one article by Abdou et al., authors found that added fat led to an early rise of blood glucose (0–3 h postprandially), that regressed after 3 h. The comparative test meal (high protein meal) caused a gradual rise of blood glucose levels in the first 3 h that peaked at 4.5 to 5 and were higher than the high fat meal [28]. This result contradicted the traditional perception that high content fat delays gastric emptying rise of blood glucose levels. Glycaemic responses to high fat meals (and low protein) have been described as initially reducing glucose levels within the first 1–2 h, followed by elevated levels of up to 5 h. These controversial findings advocate the need for further investigation into the role of fat depending on the type (e.g., triacylglycerol), amount and combination with other macronutrients.

#### 5.1.2. Protein

ISPAD gives clear recommendations for daily intake based on age (see results section) but no intake per meal [4]. No unified definition for high protein exists in literature. Definitions of high protein ranged between 40 g of protein and 60 g total [21,33,38]. In high protein only test meals Paterson et al. used 75 g in one study population and found significantly increased blood glucose levels between minutes 150 to 300 postprandially [25,40]. This suggests even longer monitoring for high protein meals [32].

With the popularity of alternative resources of protein (plant based vs. meat based) further research on protein thresholds and sources will be necessary. While low carb diets are not recommended, especially for patients with diabetes, they contain a higher percentage of protein and are becoming ever more popular. Glycaemic response to protein was reported in the results section (see above).

Bell et al. wrote that ≥230 g of a lean steak with salad may require a different insulin dosing strategy than for protein and carbohydrate meals [42]. Evans et al. found that 50% more insulin is necessary to maintain euglycaemia after a high protein meal (as defined as 60 g protein) [33].

While these statements are reasonable, the question remains which diabetic toddler/infant consumes these investigated absolute (and non-age-adjusted) amounts of fat and protein in a single serving. The use of non-adjusted meals in a high number of reviewed studies advocates the need for further paediatric studies with stratification depending on age and/or body weight. These studies may help to define age-adjusted thresholds for when macronutrients start becoming relevant when calculating bolus insulin. The existence of thresholds could further influence patient education. Not only based on levels of patient expertise, (as already proposed) [46] but also through age-stratification for insulin-algorithm and considering macronutrients as well as meal preferences.

#### 5.1.3. Mixed Meals (Fat and Protein Combined)

No unified definition could be found when researching HFHP in single meals. As presented in Table 1 and Table 2 various combinations were used to define HFHP. A relevant number of meals consumed in western societies are based on fast food and pre-prepared convenient food containing high amounts of fat and carbohydrates but also protein.

The effect of fat and protein on postprandial hyperglycaemia is additive [21]. In HFHP meals found prolonged hyperglycaemia from 3 h to 12 h depending on the observation time but with different return to baseline glucose levels results [21,32]. This reflects the fact that the more complex a meal, the longer the observation period should be.

In the literature research we identified three authors, who decided to use pizza (composition between studies and macronutrients of pizzas were non comparable) as a meal. All three studies used CSII [31,35,41]. When using CHC prolonged hyperglycaemia up to 6 h was observed [35]. When taking fat and protein into calculation, returning to baseline glucose levels was achieved but resulted in four hypoglycaemic events in 12 patients (significant at 240 min of observation time) [31]. No studies on standardized fast food with a stable macronutrient composition (e.g., BigMac^®^) were found during research.

### 5.2. Special Issues: Insulin Resistance—The Role of Puberty and Duration of Illness

While duration of illness (at least 1 year) was regularly stated, pubertal stage was not given. Study populations were almost equally distributed by gender except for one article [32]. BMI and HbA1c (see Table 1 and Table 2) reflect adherence to therapy. Short duration of illness and prepubertal stages result in a lower insulin resistance. Van der Hoogt et al. stated that the amount of insulin increased with the duration illness [27]. This is understandable as a longer duration of illness usually results in an increased insulin resistance as well as a beta cell exhaustion and therefore more significantly insulinopenia. These factors lead to higher insulin doses.

Insulin resistance is at a peak during time of puberty as well as in patients with poor diabetes management. The role of insulin resistance is insufficiently discussed in the articles. This leaves the question if results from paediatric populations are comparable to those of adult patients and what this means for mixed age study populations. The role of insulin resistance on calculating bolus insulin should be taken into account as part of the inter-individual therapy. Further studies on this point will be necessary [27].

### 5.3. The Role of Diets, Daytime of Consumption and Order of Nutrients

In the studies we read, the increased interest of children and adolescents in specialised diets (e.g., low carb diets, vegan) was discussed but not investigated. The same is true for the influence of the time of day the meal is consumed and the order in which macronutrients (e.g., desert before main course) are consumed. This is even more important for children who can be picky eaters and results in switching meals. The Grill study by Neu et al. put an emphasis on the diurnal variation in insulin sensitivity. The authors stated that consuming the same meal on various times of the day may result in different amounts of needed bolus insulin [32]. Various authors showed that consuming carbohydrates at the beginning of a meal leads to lower levels of ghrelin, shortened period of satiety and increased risk for obesity. On the other hand, consuming fat before carbohydrates leads to a delay in gastric emptying resulting in postponed elevation of glucose levels rising [1,2,3,47,54].

### 5.4. Technical Aspects—Role of MDI, CSII & CGM

While both CSII and MDI were used to administer bolus insulin, a clear preference for CSII in combination with CGM was observable (see Table 1 and Table 2) in the articles.

CSII combined with CGM is considered state of the art [55]. As it is readily available in most richer nations the tendency of study protocols to lean towards this technology leaves the question of how patients and countries unable/unwilling to use CSII and or CGM will benefit from new algorithms. In the studies investigating CSII the dual wave bolus was considered the superior method for maintaining postprandial euglycemia after HFHP meals. There is no unified recommendation on the amount of bolus insulin or the details of the dual wave bolus (split-percentage and duration) [49].

In conventional MDI therapy with finger prick single point measurements the dynamic of prolonged elevation of glucose levels may be missed. Therefore, CGM can help identify prolonged postprandial hyperglycaemia better. This aids individual needs for adaptation of bolus insulin, both in dosage and algorithm for MDI and CSII. It should further lead the user (i.e., patient) to self-reflect on the impact of macronutrients and modify his therapy thereafter. If a CGM is not available/not wanted, blood glucose after a HFHP meal should be measured for a longer period of time. Further studies need to focus on how to best apply (duration and frequency) single point measurements after a HFHP meal. In all original articles with HFHP additional insulin for a HFHP combination was necessary.

### 5.5. Current Approaches to Estimate Bolus Insulin

While all reviewed articles (see Table 1 and Table 2) agree that additional bolus insulin is necessary for covering protein and/or fat in meals, they do not agree on a threshold to take these macronutrients into account.

Results for additional insulin compared to CHC varied widely. Smith et al. recommended 40% additional bolus for CSII insulin and 25% additional bolus insulin in MDI [37,39].

In all studies for MDI we found no more than 160% of CHC-calculated bolus insulin. Smith et al. justified this percentage by arguing that 160% is what Pankowska equation would result in [39]. As previously stated Pankowska equation was linked to increased hypoglycaemia by some authors [10,30,35,38].

This leads to the question if modifying Pankowska equation in further studies should be attempted. For this, three points need to be considered. First, which macronutrients need to be taken into consideration? All reviewed authors (see Table 1 and Table 2) conclude that carbohydrates and protein are relevant for bolus insulin. Some authors like Schweitzer et al. argue that fat can be left out [36]. In the studies on high fat meals the last statement is clearly contradicted. The second point is if all fat and protein need to be considered or if thresholds for a single macronutrient or the combination exist. Suggestions for protein rich only meals exist as stated in the results section. No such thresholds were given for high fat and HFHP meals. Third, a correction of the amount of insulin administered seems reasonable as increased rates of hypoglycaemia were reported [10,30,35,38]. Additional bolus insulin between 25% and 40% for HFHP meals was reported, which is well below the additional 60% when using Pankowska equation [39]. This contradicts findings by Lopez et al. who found that the insulin dose was about 17% higher for high fat (HF) meals and 24% higher for high protein (HP) meals compared to CHC when using Pankowska equation but they used no more than 40% [29]. This resulted in better glycaemic control and a better safety profile. Simply adding insulin to a CHC based insulin dose may be simple but is not representative of reality. In everyday use and patient education taking fat and protein into consideration may be more sophisticated. Pankowska et al. are correct in using fat and protein units to calculate insulin. The equation is elegant and usable. Stated risks of hypoglycaemia cannot be denied but may be improved. In the future, studies with a bigger study population may lead to modifications of the equation or a completely new one. The risk of hypoglycaemia could further be reduced when used closed loop systems that suspend insulin before hypoglycaemia.

## 6. Gaps of Research & Outlook

During the review process of the articles the following gaps of research and future needs became apparent.

### 6.1. Study Design

#### 6.1.1. Gaps

Currently there are only a small number of studies with very low numbers of paediatric participants. These studies are heterogenous and the majority of these studies consist of a mixed-study population (adults and paediatric population). Parameters for study meals, glucose monitoring, duration and outcome differ vastly.

There is no unified complex test meal adapted to age/body weight for studies.

#### 6.1.2. Future Needs

Studies with a large number population (paediatric only).

Expansion of observation time of 12 h and even longer seems reasonable based on the current results. CGM should be routinely used as a time in range reflects the glucose levels better than single-point measurements [13]. Studies should further use a stratified approach (e.g., age, gender, pubertal stage, duration of diabetes, BMI, HbA1c and kind of treatment (CSII vs. MDI)).

Further research on the amount, quality and intake-order of macronutrients and the role of daytime of a meal on postprandial glucose levels. Further studies regarding the influence of duration of illness and insulin resistance. No insulin clamp study for fat currently exists.

### 6.2. Nutritional Key Points

#### 6.2.1. Gaps

Unified definitions what high fat and/or high protein in a meal means.

A clear threshold of when fat and/or protein must be taken into calculation for bolus insulin.

#### 6.2.2. Future Needs

Recommendation of food composition for a diabetic’s single meal, as only a daily recommendation of macronutrient content exists.

### 6.3. Monitoring of Glucose Levels

In studies: Use of CGM to provide glucose -AUC/TIR for at least 12 h postprandially

In everyday life: providing patients with CGM if possible/desired by patient. In everyday life: If single-prick measurement is the existing monitoring a clear recommendation on postprandial measurements (frequency and duration) has to be given.

### 6.4. Administering of Bolus Insulin

Development of a safe and easy to use algorithm for bolus insulin. This should reflect carbohydrates, fat, and protein as well as inter-individual needs. Needs to be usable by CSII and MDI patients alike, independent of CGM-use. This could include a modification of the Pankowska equation to reduce the risk of hypoglycaemia.

### 6.5. Special Issues

Improved patient education models that include fat and protein are essential in the improvement of glycaemic control. Only if the patient understands the effects of nutritional (high) fat and (high) protein an optimal and individualized therapy can be conducted.

## 7. Conclusions

The search of current literature resulted in a limited number of publications (n = 28) investigating the role of nutritional (high) fat and/or (high) protein in children with T1DM. These studies proved to be highly heterogeneous and contained only a limited number of paediatric participants. The research showed that there is no unified definition of HF/HP.

The role of protein and fat as macronutrients in children with diabetes has been recognized, yet CHC (not taking fat and protein into account) is still the dominant form of calculating insulin boli. This however leads to prolonged hyperglycaemia and unsatisfying results when it comes to AUC/TIR. Improving AUC/TIR leads to reducing long term complications of T1DM.

Although methods for calculating additional insulin for HF/HP meals have been developed (e.g., Pankowska equation and Food Insulin Index), they are impractical in daily use or offer a higher risk of hypoglycaemia. Therefore, an easy to use, inter-individual algorithm for bolus insulin covering HF/HP is necessary (e.g., modifying Pankowska equation or creating a new one). This emphasizes the future need for tailored therapy regimen, improved patient education on macronutrients and if possible, usage of CGM. Improved therapy and education that are implemented early in a patient remain for the rest of life.

Concerning further studies: a clear definition of HF/HP is inevitable for comparison.

Further studies are necessary due to a rising prevalence of T1DM in children and the technical advantages of CGM and AUC/TIR as the “new” HbA1c. These studies should include a greater number of participants and focus of stratification (age, gender etc.), unified definition of HF/HP and even consider developing a standardized mixed meal test to facilitate individual therapy.

A one fits all therapy-approach for children with T1DM is outdated and the future clearly lies in tailored therapy emphasizing the role of macronutrients and the role of nutrition itself. Due to the incomparability of studies and the low number of study participants, study findings were inconsistent regarding the role of dietary fat and protein for prandial insulin requirements in children with type 1 diabetes. The conclusion remains that high fat and/or high protein meals require more bolus insulin than low fat/low protein meals with the identical amount of carbohydrates [42].
